# Visual Foraging With Fingers and Eye Gaze

**DOI:** 10.1177/2041669516637279

**Published:** 2016-03-17

**Authors:** Ómar I. Jóhannesson, Ian M. Thornton, Irene J. Smith, Andrey Chetverikov, Árni Kristjánsson

**Affiliations:** Faculty of Psychology, University of Iceland, Iceland; Department of Cognitive Science, University of Malta, Malta; Faculty of Psychology, University of Iceland, Iceland; Faculty of Psychology, University of Iceland, Iceland; Department of Psychology, Saint Petersburg State University, Saint Petersburg, Russia; Cognitive Research Lab, Russian Academy of National Economy and Public Administration, Moscow, Russia; Faculty of Psychology, University of Iceland, Iceland; Institute of Cognitive Neuroscience, University College London, UK

**Keywords:** visual foraging, finger foraging, eye gaze foraging, attention, visual attention, visual search

## Abstract

A popular model of the function of selective visual attention involves search where a single target is to be found among distractors. For many scenarios, a more realistic model involves search for multiple targets of various types, since natural tasks typically do not involve a single target. Here we present results from a novel multiple-target foraging paradigm. We compare finger foraging where observers cancel a set of predesignated targets by tapping them, to gaze foraging where observers cancel items by fixating them for 100 ms. During finger foraging, for most observers, there was a large difference between foraging based on a single feature, where observers switch easily between target types, and foraging based on a conjunction of features where observers tended to stick to one target type. The pattern was notably different during gaze foraging where these condition differences were smaller. Two conclusions follow: (a) The fact that a sizeable number of observers (in particular during gaze foraging) had little trouble switching between different target types raises challenges for many prominent theoretical accounts of visual attention and working memory. (b) While caveats must be noted for the comparison of gaze and finger foraging, the results suggest that selection mechanisms for gaze and pointing have different operational constraints.

## Introduction

Imagine yourself on a crowded sidewalk. You are about to feed coins into a parking meter. You drop your wallet and coins of various sizes and denominations scatter around you. You collect the coins as quickly as you can while avoiding irrelevant stimuli such as chewed gum, pieces of paper, and other debris. This is an example of a visual *foraging* task where the relevant visual features vary between the items of interest. Your visual attention is tuned to items containing the relevant features, the size, sheen, and texture of the coins.

Visual attention enables us to select relevant items for processing (Bundesen & Habekost, 2008; Pashler, 1998). A popular way of modeling visual attention involves visual search for single targets (Kristjánsson, 2006; Nakayama & Martini, 2011; Wolfe, 1998). Observers determine whether the target is present or absent (Treisman & Gelade, 1980). But for many scenarios, a search where a single decision is made, and the search then ends, may not be very realistic. This may work as an analogy for when you search for your car keys, but as we interact with our environment our goals may not necessarily be so narrow as to involve one single target. Multiple-target foraging may better tap into the nature of attentional allocation across the visual field (Cain, Vul, Clark, & Mitroff, 2012; Gilchrist, North, & Hood, 2001; Hills, Kalff, & Wiener, 2013; Kristjánsson, Jóhannesson, & Thornton, 2014; Wolfe, 2013).

### Finger Foraging

Recently, we introduced a new “finger foraging” task to explore the behavior of human participants when faced with search for multiple items from more than one target category (Kristjánsson et al., 2014). Directly inspired by the seminal foraging work of Dawkins (1971), our displays consisted of 40 target items (e.g., 20 red and 20 green dots) interspersed with 40 distractor items (e.g., 20 yellow and 20 blue dots). The task was to cancel all target items as quickly as possible by tapping them without touching any distractor items.

When individual targets were defined by a single feature (i.e., color), participants selected randomly from the two target categories. When target categories were defined as conjunctions of color and shape (e.g., red circles and green squares amongst red squares and green circles), most participants selected items in long “runs” of the same type (Kristjánsson et al., 2014). A “run” in this context refers to the selection of targets of the same type in nonrandom sequences that are longer than would be expected by chance.

To our knowledge, this was the first demonstration of attention-modulated, run-like behavior in humans. The close parallel with animal studies, where foraging behavior can switch from random selection amongst all available sources when food is conspicuous to run-like behavior when it is cryptic (Bond, 1983; Bond & Kamil, 2006; Cooper & Allen, 1994; Dawkins, 1971; Heinrich, Mudge, & Deringis, 1977; Jackson & Li, 2004; Kono, Reid, & Kamil, 1998; Langley, Riley, Bond, & Goel, 1995; Pietrewicz & Kamil, 1977), led us to suggest that common attentional constraints might mediate search behavior across a broad range of species (Dukas, 2002; Dukas & Ellner, 1993).

### Contrasting Finger and Eye Gaze Foraging

One of the central aims of this paper is to contrast foraging with fingers, as above, to foraging by gaze. A common conception of motor control is that the movement of eye or hand to a particular location involves a similar attention plan (Deubel & Schneider, 2004; Rizzolatti, Riggio, Dascola, & Umiltá, 1987). Consistent with this, Reyes-Puerta, Philipp, Lindner, and Hoffmann (2010) found neurons in the superior colliculi (SC) of rhesus monkeys that coordinate eye and hand movements. Furthermore, Hagan, Dean, and Pesaran (2012) observed common neuronal activity in the lateral intraparietal area in macaque monkeys, when eye and hand movements are coordinated to targets at the same locations. There is also evidence for a neural pathway that links eye and limb movements together in response to suddenly appearing task-relevant stimuli (Pruszynski et al., 2010).

But other studies suggest that things are not this clear-cut. Linzenbold and Himmelbach (2012) reported that gaze and hand control are dissociated in human SC. The SC also contains neurons that respond when monkeys touch an object with their hands, but are silent when the monkeys only look at the objects (Nagy, Kruse, Rottmann, Dannenberg, & Hoffmann, 2006). Furthermore, there is evidence for both gaze-independent reach neurons and gaze-related reach neurons in the SC (Lünenburger, Kleiser, Stuphorn, Miller, & Hoffman, 2001).

Another reason for comparing gaze and finger foraging is the hypothesized relation between eye movements and visual attention (Deubel & Schneider, 1996; Hoffman & Subramaniam, 1995; Kowler, Anderson, Dosher, & Blaser, 1995; Kristjánsson, 2007, 2011; Kristjánsson, Chen, & Nakayama, 2001; Kustov & Robinson, 1996). While many studies show that similar relations hold for attention and finger control (Bekkering & Neggers, 2002; Deubel & Schneider, 2004; Eimer, Van Velzen, Gherri, & Press, 2006; Schiegg, Deubel, & Schneider, 2003), Jonikaitis and Deubel (2011) argued that attentional resources are allocated independently to eye and hand movement targets, suggesting that the goals for the two are selected by separate mechanisms.

### Current Goals

We had three main goals in the current study. First, using identical displays, we wanted to replicate the pattern of finger foraging seen in our previous study. Second, we wanted to extend these findings by examining a different response modality where observers canceled predesignated targets by fixating them for 100 ms. Our primary concern was whether run-like behavior would be observed with gaze foraging. We note that we did not attempt to fully equate the task parameters for the two modalities (see “Methods” section for details). Our primary focus, then, will be on within-modality patterns of foraging. However, we do provide both qualitative and quantitative across-modality comparisons for the sake of completeness. Comparing finger and gaze foraging in the same individuals could shed important light on the nature of the attentional constraints operating in the two versions of the task.

Thirdly, we wanted to explore another aspect of our original data: the presence of clear individual differences in how attention constrains search. Specifically, 4 of our original 16 participants showed essentially no change in finger foraging behavior between feature and conjunction conditions. We termed them “super-foragers” as their continued random selection from both categories during conjunction search allowed them to complete the task more efficiently—that is with less overall movement—with only very modest time cost (Kristjánsson et al., 2014; see Watson & Stayer, 2010 for related findings). We were particularly interested in whether “super foraging” behavior would be observed during finger and gaze foraging with the current sample of participants and if so, how stable it would be across individuals. As we highlight in the “Discussion” section, such immunity to clear increases in attentional load would raise interesting questions for current theories of attention and working memory (WM).

## Methods

### Participants

Twenty-one naïve observers with normal or corrected to normal vision (15 males; 22 to 50 years old, *M* = 26.9 years, *SD* = 6.5 years, two left-handed) participated. Five were excluded since their data were incomplete.

### Apparatus

The finger foraging stimuli were displayed on an iPad with screen dimensions of 20 × 15 cm and a resolution of 1024 × 768 pixels. The iPad was placed on a table in front of the participants in landscape mode (viewing distance ≈ 50 cm). Stimulus presentation and response collection were carried out with a custom iPad application written in Objective-C using the Xcode and Cocos2d libraries.

For gaze foraging, a high-speed eye-tracker from Cambridge Research Systems tracked observers’ dominant eye at 250 Hz (spatial accuracy 0.125°–0.25°). Stimuli were displayed on a 100 Hz 19″ Hansol CRT screen (model: 920D resolution: 1024 × 768) controlled by a 2.33 GHz PC (Windows 7; RAM = 4 Gb). Viewing distance was 60 cm (ensured with head rest). The experimental program was written in Matlab and functions from the Psychtoolbox (Brainard, 1997; Kleiner et al., 2007; Pelli, 1997) and the eye-tracker toolbox (Cambridge Research Systems, 2006) were used to control stimulus presentation and data collection.

### Stimuli

**Figure 1.**
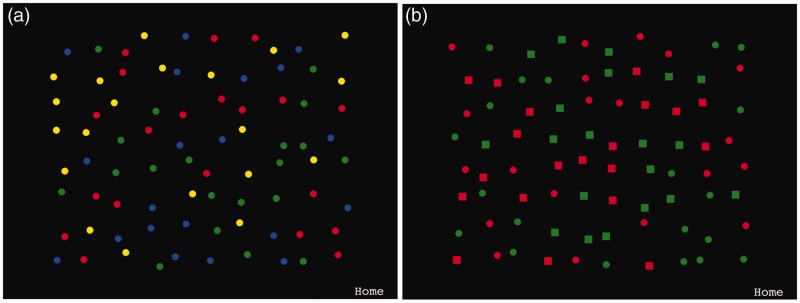
The experimental stimuli. (a) The stimuli in the feature foraging condition. The targets to cancel were either all the red and green or blue and yellow items. (b) The stimuli in the conjunction foraging condition. The targets were either all red disks and green squares, or all green disks and red squares.

For feature foraging, the targets and distractors were either red or green disks among blue and yellow distractor disks, or vice versa (see Figure 1). For conjunction foraging, the targets and distractors were either red squares or green disks among red disks and green squares, or vice versa. The stimuli were distributed randomly across a virtual grid on a black background but their positions were adjusted through a random offset in both the vertical and horizontal directions for heterogeneous appearance.

For finger foraging, there were 40 targets and 40 distractors with a diameter of 20 pixels that were distributed across a 10 × 8 grid offset from the edges of the screen by 150 × 100 pixels with the viewing area occupying 15 × 12 cm. Minimum gaps between stimuli prevented overlap. For gaze foraging, there were 16 targets and 16 distractors. All had a diameter of 1°, distributed randomly across the screen but offset from its edges by 3.2°. If observers’ gaze fell within a square region of interest (ROI) surrounding each stimulus, they were considered to be fixating that stimulus. Note, importantly that the target ROI was 4° but 1° around the distractors to minimize accidental selection of distractors. The fixation time required for selection was 100 ms (after which the target disappeared). Feature versus conjunction foraging and gaze versus finger foraging were administered in counterbalanced order. Stimulus categories for the foraging types were consistently paired within participants and across foraging method although they were randomized and counterbalanced *between* participants.

### Analysis

Our primary dependent measure was average run length on a given trial. Average run length is a good indicator of foraging strategy—short runs suggest random target selection, longer runs suggest attention-constrained foraging—and is simply computed by summing the length of consecutive choices of the same target and dividing by the total number of runs on a given trial. We compared within modality run length in the feature and conjunction conditions, averaged across-trial, with paired *t* tests.

In addition to raw run length data, we also computed normalized scores to aid comparison across modalities. We subtracted individual trial averages from a grand mean, computed across both feature and conjunction conditions, dividing this value by the overall standard deviation, again computed across the two conditions. These normalized scores were compared using a 2 (Condition: feature, conjunction) × 2 (Modality: finger, gaze) repeated measures analysis of variance (ANOVA). To assess whether observers showed differences between feature and conjunction foraging, we categorized them into same-pattern or different-pattern groups based on whether there was more than a one standard deviation difference between their standardized score for the two conditions. We also measured completion time, total movement length, and error rates. These remaining dependent variables are described in more detail in the relevant results sections.

### Procedure

In a sound-proof booth, observers completed the tasks by fixating (or tapping) all targets while avoiding distractors. When a trial was completed, a message conveying successful completion appeared on the screen followed by the subsequent trial. If a distractor was selected, an error message appeared and a new trial started. Finger foraging was performed under normal illumination. Participants performed five practice trials and then had to complete 20 trials of each type correctly (only those trials were analyzed). During gaze foraging, the only lighting came from the computer monitors except that three participants required mild background lighting for accurate eye-tracking due to enlarged pupils in the dark. After calibration, participants performed 10 practice trials followed by the 20 experimental trials.

## Results

### Run Length

**Figure 2.**
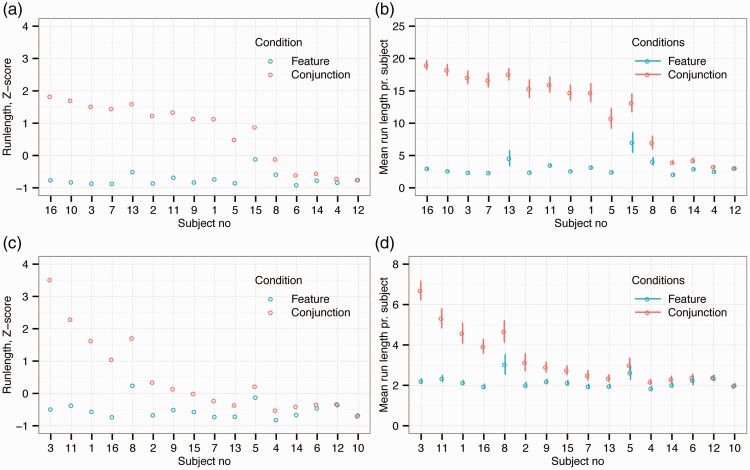
The results from the finger and gaze foraging experiments. (a) Normalized run length (z-scores) and (b) raw run length for each observer during finger foraging. (c) Normalized run length (z-scores) and (d) raw run length for each observer in the gaze foraging condition. In all panels, observers are rank ordered on the abscissa by difference in performance between feature versus conjunction foraging.

Figure 2(a) presents the average normalized run length (and Figure 2(b) the average raw run length) for individual observers in the finger foraging condition. The results are ordered by performance difference between feature and conjunction foraging by observer. As in our previous study, there was a reliable difference in run length between feature and conjunction foraging. Specifically, the average run length was significantly shorter (paired *t*(15) = 6.19, *p* < .001, Cohen’s *d* = 1.75) during feature (*M* = 3.2 run, SD = 2.4 run) than conjunction (*M* = 12.7 run, *SD* = 7.3 run) foraging. Within this overall pattern, however, there are clear individual differences. Classifying observers in terms of the distance between their standardized scores in the two conditions, as described earlier, revealed that 11 participants showed consistent differences between feature and conjunction foraging, while 5 did not (see Figure 2(a) and (b)).

**Figure 3.**
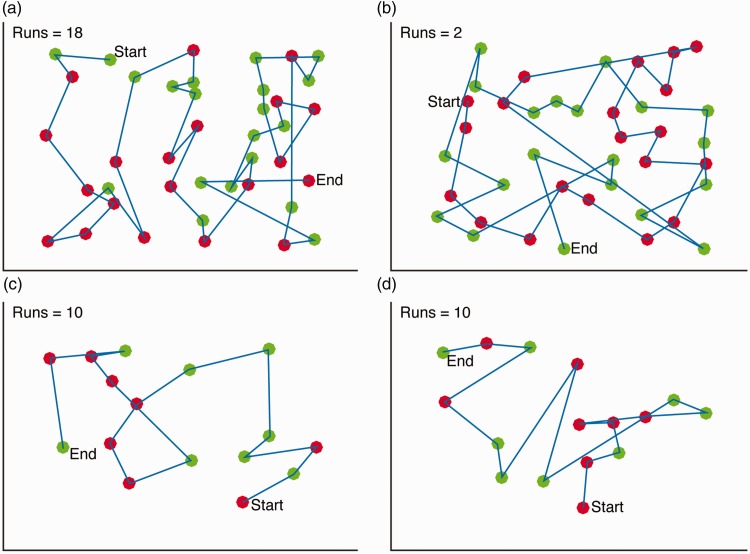
Randomly selected foraging paths. The figure shows typical foraging paths for finger and gaze foraging separately for the feature and conjunction condition. The number of runs in each condition is also shown: (a) Finger feature foraging; (b) Finger conjunction foraging; (c) Eye feature foraging and (d) Eye conjunction foraging.

Similarly, Figure 2(c) shows normalized run length data (and Figure 2(d) the raw run length) for gaze foraging. As for finger foraging, there was a significant difference in run length between the two conditions. Again, the average run length was significantly shorter (paired *t*(15) = 3.67, *p* = .002, Cohen’s *d* = 0.74) for feature (*M* = 2.3 run, *SD* = 0.9 run) than conjunction foraging (*M* = 3.4 run, *SD* = 1.9 run). However, as can be seen by comparing Figure 2(a) and (c), the separation between the two conditions appears much less marked for gaze foraging. This impression was confirmed when classifying individual observers, as with gaze foraging only 5 participants had consistent differences between feature and conjunction conditions, while the remaining 11 did not. Figure 3 shows example foraging paths for finger and gaze foraging.

Below, we more directly compare performance in finger and gaze foraging. As already noted, such comparisons need to be interpreted with caution, given the methodological differences between the tasks, but are nonetheless useful as exploratory steps. First, we made a direct quantitative comparison on the normalized run length data. A 2 × 2 repeated measures ANOVA on run length revealed a significant main effect of condition (feature versus conjunction; *F*(1, 15) = 48.8, *p* < .001; *η*_partial_squared_ = 0.76) and of foraging measure (finger versus eye gaze; *F*(1, 15) = 21.0, *p* < .001; *η*_partial_squared_ = 0.58) but also a highly significant interaction (*F*(1, 15) = 23.6; *p* < .001; *η*_partial_squared_ = 0.61). This is highlighted by comparing the number of participants classified as having the same or different patterns of foraging across feature and conjunction conditions. This difference in proportions between finger (5/16; 31.25%) and gaze (11/16; 68.75%) foraging clearly indicates that a larger number of participants continued to use random category selection when using their eyes.

Second, we computed the number of trials classified as nonrandom for each observer and compared these with a 2 × 2 ANOVA. To classify a trial as nonrandom, we used One-Sample Runs Tests with a Bonferroni correction to adjust the level of alpha for multiple tests (see Kristjánsson et al., 2014 for details). Table 1 provides a summary of this classification. We found a main effect of condition (*F*(1, 15) = 63.8; *p* < .001; *η*_partial_squared_ = 0.81) of foraging measure (*F*(1, 15) = 115.7; *p* < .001; *η*_partial_squared_ = 0.89) and a significant interaction between the two factors (*F*(1, 15) = 42; *p* < .001; *η*_partial_squared_ = 0.74). Most importantly, these results show that there is very little nonrandom foraging with gaze while for conjunction foraging with fingers the majority of trials are nonrandom consistent with the fact that we see very long runs in that condition.

**Table 1.** Number of Trials Classified as Nonrandom as a Function of Participant, Foraging Method, and Condition.

	Finger foraging		Gaze foraging	
Participant no.	Feature condition	Conjunction condition	Feature condition	Conjunction condition
1	6	19	0	7
2	0	20	0	3
3	0	20	0	14
4	2	6	0	0
5	1	15	1	2
6	0	11	1	0
7	0	20	0	1
8	8	18	3	7
9	1	20	0	2
10	1	20	0	0
11	12	20	0	9
12	4	4	0	0
13	5	20	0	0
14	4	13	0	0
15	7	20	0	2
16	6	20	0	2
Average	3.6	16.6	0.3	3.1
*SD*	3.4	5.2	0.8	4.0

*Note.* A trial is classified as nonrandom if it deviates significantly from the expected number of runs (assessed with a one-sample runs test). For example, many trials in the finger foraging conjunction condition are classified as nonrandom as participants typically use only two very long runs.

**Figure 4.**
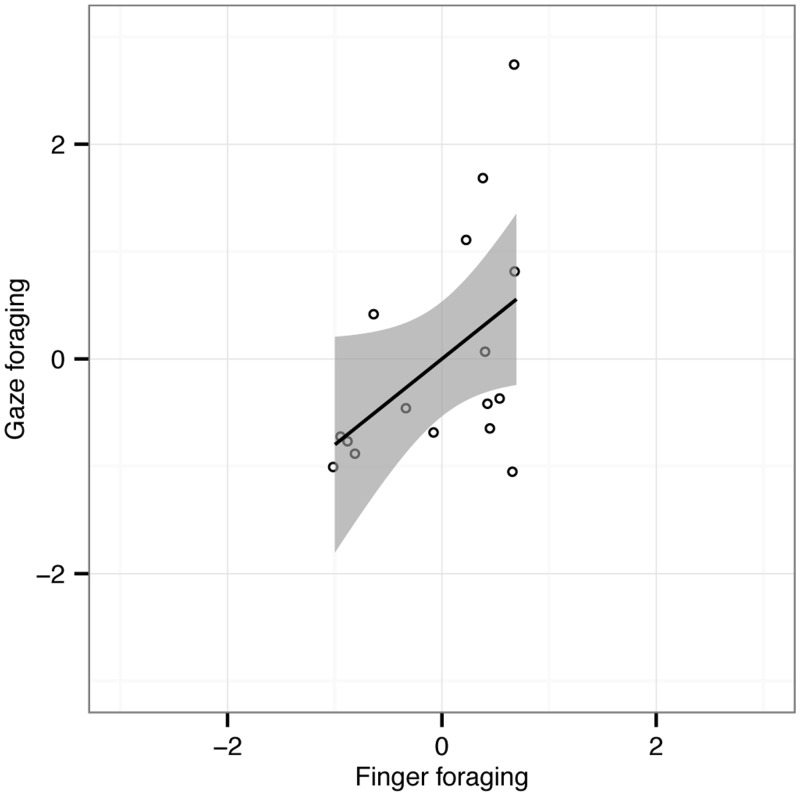
Scatterplot showing normalized differences of mean run length between feature and conjunction foraging for gaze foraging (ordinate) and finger foraging (abscissa) for the 16 observers individually. The Pearson correlation (*r*) was .47 (*p* = .032, one-tailed). Shaded areas represent 95% CI of the linear fits to the data.

Finally, we explored whether individual participants had similar run length behavior in the finger and gaze foraging conditions. Correlations on the difference scores (conjunction—feature) suggest that participants with similar feature and conjunction performance in the finger foraging task also have a tendency towards similar differences for gaze foraging (*r* = .47; *p* = .032; see Figure 4). This correlation is far from perfect, however.

In summary, across a number of comparisons, we observe smaller differences between feature and conjunction foraging for gaze foraging than finger foraging. Gaze foraging therefore appears not to be under such strong constraints as finger foraging when the same attentional load is applied, at least for the displays tested here.

### Switch Costs

**Figure 5.**
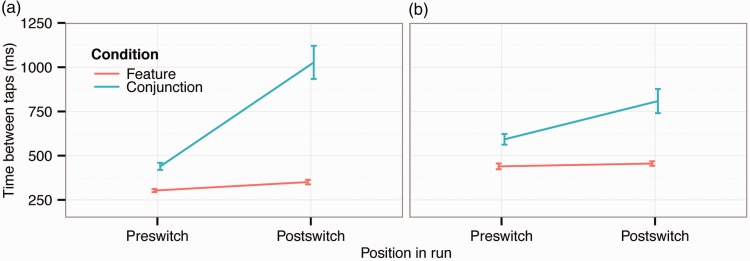
Response time switch costs between taps during finger and gaze foraging. The error bars show ±1 *SEM* based on within-subject variance: (a) Finger and (b) Gaze.

**Figure 6.**
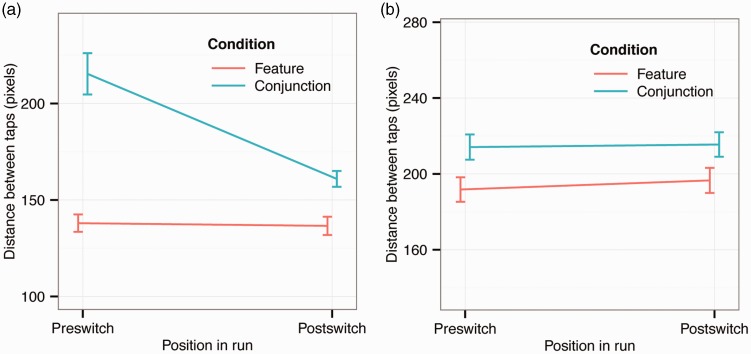
Distance between consecutive taps as a function of whether observers switched between target types or not during foraging. The error bars show ±1 *SEM* based on within-subject variance: (a) Finger foraging and (b) Gaze foraging.

Figure 5 presents switch costs within trials that measure whether there is a difference in movement time from the last target to the next as a function of whether observers switch between target types or continue choosing the same target. Figure 6 shows switch costs in distance between consecutive taps (as in Figure 5). Switch costs are overall higher in the conjunction condition, but consistent with the results on run length, switch costs during conjunction foraging are much larger for finger than gaze foraging. Again there is a large difference between finger and gaze foraging, perhaps reflecting differences between the mechanisms involved in the two foraging types.

A three-way repeated measures ANOVA on response time switch costs (Figure 5) revealed significant main effects of condition (conjunction vs. feature; *F*(1, 15) = 100, *p* < .001; *η*_partial_squared_ = 0.87) and switching (*F*(1, 15) = 52.4, *p* < .001; *η*_partial_squared_ = 0.78) but not of foraging method (*F*(1, 15) = 2.05, *p* = .17; *η*_partial_squared_ = 0.12). The two-way interactions between condition and switch (*F*(1, 15) = 45.2, *p* < .001; *η*_partial_squared_ = 0.75), condition and foraging method (finger vs. gaze; *F*(1, 15) = 8.45, *p* = .011; *η*_partial_squared_ = 0.36) and switch and foraging method (*F*(1, 15) = 23.4, *p* < .001; *η*_partial_squared_ = 0.61) were all significant. Finally, the three-way interaction was significant (*F*(1, 15) = 17.8, *p* < .001; *η*_partial_squared_ = 0.54), confirming that switch costs as a function of feature versus conjunction foraging differ between the two foraging methods.

A three-way repeated measures ANOVA on switch costs in distance between consecutive taps (Figure 6; note difference in scales between conditions) showed significant main effects of condition (*F*(1, 15) = 77.5, *p* < .001; *η*_partial_squared_ = 0.838), of switching (*F*(1, 15) = 24.7, *p* < .001; *η*_partial_squared_ = 0.623), and of foraging method (*F*(1, 15) = 41.1, *p* < .001; *η*_partial_squared_ = 0.733). The two-way interactions between condition and foraging method, between condition and switch, and between foraging method and switch were all significant (*F*(1, 15) = 16.3, *p* = .001, *η*_partial_squared_ = 0.521; *F*(1, 15) = 39.6, *p* < .001, *η*_partial_squared_ = 0.725; *F*(1, 15) = 36.4, *p* < .001, *η*_partial_squared_ = 0.708, respectively). The three-way interaction was also significant (*F*(1, 15) = 15.3, *p* = .001, *η*_partial_squared_ = 0.505). The most notable result (highlighted in Figure 6) is that during conjunction foraging with fingers, observers have a strong tendency to choose the same target as on the last trial, and they will “travel” far in the display to choose such a target, presumably not choosing closer targets of the other type. Such a difference is not seen for gaze foraging.

### Finishing Time and Traveling Distance

For finger foraging, the average finishing time for each trial was 12.6 s for the feature condition and 17.6 s for the conjunction condition (paired *t*(15) = 6.5, *p* < .001). This indicates that conjunction foraging was, on average, more difficult than feature foraging. The average traveling distance for each trial was 5346 pixels during feature foraging and 6436 for conjunction foraging (paired *t*(15) = 8.8, *p* < .001). This is not surprising since if observers use longer runs of foraging the same target, they will by necessity travel longer. The gaze foraging data mirror the finger foraging data: Average finishing time for each trial was 6.7 and 10.2 s for feature and conjunction foraging, respectively (paired *t*(15) = 6.9, *p* < .001). For feature foraging, the average traveling distance was 2922 and 3261 pixels for conjunction foraging (paired *t*(15) = 4.2, *p* < .001).

### Foraging Organization

**Figure 7.**
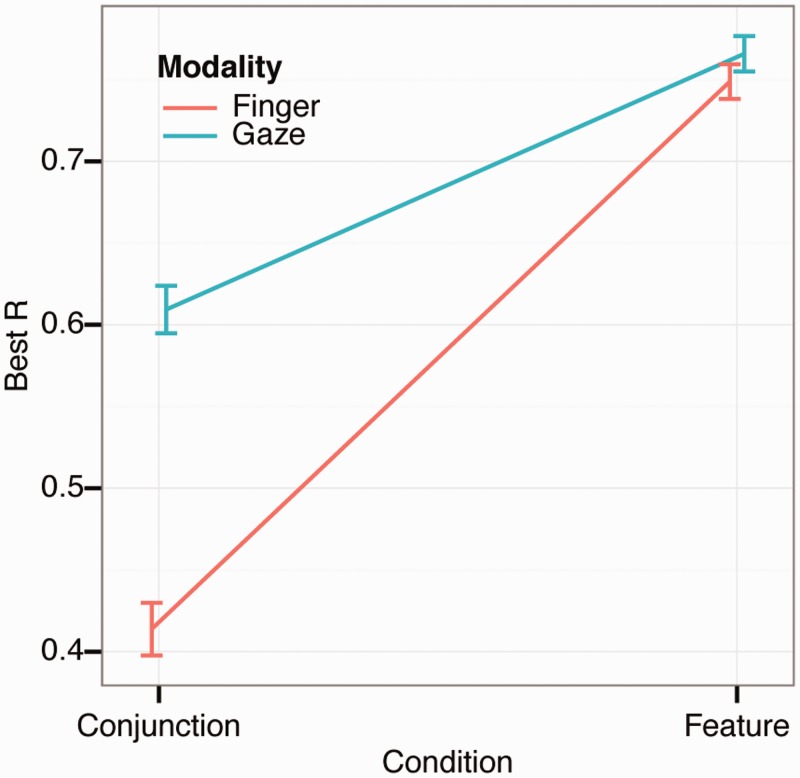
Foraging organization (as measured with best *R*) during finger and gaze foraging. The error bars show ±1 *SEM* based on within-subject variance.

Finally, we analyzed foraging organization. Calculating the correlation between the Cartesian coordinates of the targets and the sequence of how the targets are selected provides information on search organization (Woods et al., 2013). A high correlation between x-coordinates and selection sequence suggests that foraging was performed with horizontal sweeps across the search space. Similarly, a high correlation between the y-coordinates and the selection sequence suggests that participant foraged in vertical sweeps. If the correlation is low, the foraging is disorganized. The highest correlation (irrelevant of axis) is the Best R and yields an estimate of the degree to which foraging was organized (shown in Figure 7). A two-way repeated measures ANOVA revealed a significant main effect of condition (*F*(1, 15) = 42.3, *p* < .001, *η*_partial_squared_ = 0.74) and of foraging method (*F*(1, 15) = 12.1, *p* = .002, *η*_partial_squared_ = 0.45) and a significant interaction (*F*(1, 15) = 22.3, *p* < .001, *η*_partial_squared_ = 0.60). A post-hoc test showed that the differences were always significant except between foraging methods in the feature condition. Overall, foraging appears to be highly organized during feature foraging, indicating that participants utilize consistent horizontal or vertical sweeps through the display when attentional load is low. Such tendencies are generally reduced during conjunction foraging, but the drop is much more marked for finger foraging. This differential pattern of organization is again suggestive that the conjunction manipulation has less of an impact on eye foraging than it does on finger foraging.

### Error-Rates

Error-rates were defined as the proportion of total number of incorrect taps or fixations divided by the total number of targets. Using Wilcoxon signed-rank tests, no significant differences (*p* = .426) in error-rates between feature (Median = .014, range .003–.07) and conjunction (Median = .019, range 0–.065) conditions were found for finger foraging, but for gaze foraging the error-rates in the conjunction task (Median = .084, range .013–.094) were significantly higher (*p* < .001) than in the feature task (Median .013, range 0–.031). While significant, a difference of only 6 percentage points is unlikely to account for the strong difference in the observed foraging patterns. Specifically, there was little or no nonrandom gaze foraging while for finger foraging the majority of trials were nonrandom. If the gaze pattern reflected a speed–accuracy trade-off, the difference in error rates should be far larger.

## Discussion

We replicated our previous results on finger foraging (Kristjánsson et al., 2014) where most observers show a strict dissociation between performance during feature versus conjunction foraging—switching easily between targets when a single feature separates the two target types from the two distractor types, but staying with target types for long runs of adjacent trials during conjunction foraging. But, again, we found a subset of observers, that we had labeled “super foragers”, who did not show this pattern, but rather continued to switch categories easily on conjunction trials.

Our main new finding is that during gaze foraging, the proportion of observers who continue to switch categories is much higher than for finger foraging. While we did observe some run-like behavior for gaze foraging, very few trials across all participants were classified as nonrandom (Table 1), in comparison to finger foraging. This suggests that the mechanisms for target selection for gaze and finger pointing do not show complete overlap. In addition, the correlation between gaze and finger foraging is far from perfect, suggesting that “super-foraging” may not be a fixed trait, but may vary with task.

That gaze foraging is less constrained by the conjunction manipulation than finger foraging, is further supported by lower switch costs between target types and patterns of search organization. This may indicate that observers are simply more adept at switching between templates when eye gaze is involved, perhaps because eye gaze is a more basic, less complex behavior than finger movement (e.g., Jóhannesson, Ásgeirsson, & Kristjánsson, 2012; Leigh & Zee, 2011), and may therefore be more resistant to increased selection complexity.

Such basic differences between the two modalities could also constrain the accuracy of individual target selection events, which in turn could influence the overall pattern of run-like behavior. If participants need to allocate more resources to localize accurately when using the fingers, they may have less available capacity to aid in category switching. More generally, individual target selection criteria could interact with our more global attentional manipulation. For example, one possibility is that *within* a given modality, “super-foragers” allocate less resources to achieve precise localization, thus allowing them to more easily switch target categories under conjunction conditions. Our current data do not allow us to directly explore the link between target selection criteria and run-like behavior, but this is clearly an interesting avenue for future studies.

Finally, we note again, that the overall differences between our gaze-dependent and iPad displays, together with the possible target selection criteria issues just mentioned, made it impossible to completely equate gaze tasks and finger tasks in the current study. A caveat must therefore be noted regarding our findings, and the conclusions here need to be verified in future research. We believe, however, that such superficial differences in display and task parameters are unlikely to completely account for the lack of overlap between the two foraging modalities.

### Theoretical Implications

Critical aspects of the current results raise problems for prominent theoretical accounts of attention. This is true for the finger foraging results, but even more so for the results from the gaze foraging task. A longstanding debate in the literature on visual attention involves the nature of WM representations and how they guide attention (Awh & Jonides, 2001; Chun, Golomb, & Turk-Browne, 2011; Gazzaley & Nobre, 2012). A common assumption is that during visual search, a template of the target is loaded into memory (Bundesen, 1990; Treisman & Gelade, 1980). This requires attentional effort, especially if the template is defined by a conjunction of features, and switching between templates is similarly effortful. According to Treisman’s & Gelade’s (1980) Feature Integration Theory, binding features requires attentional effort. A reasonable strategy therefore involves sticking to the same target type during foraging. The rapid switching observed during conjunction foraging is inconsistent with this and causes problems for Feature Integration Theory and related theories that incorporate many similar concepts.

Olivers, Peters, Houtkamp, and Roelfsema (2011) proposed that only one WM representation functions as an attentional template at any time having direct access to perception (see also Van Moorselaar, Theeuwes, & Olivers, 2014). According to this, only a single WM representation controls attention at a given moment. The Boolean-map theory of attention (Huang & Pashler, 2007) makes a similar claim; that the visual input can be subdivided into to-be-attended and to-be-ignored regions on the basis of just one feature value. According to both of these theories, only one control signal at a time can be sent from WM processes to attentional mechanisms that implement visual selection. These conceptions therefore clearly predict that observers would stick to the same target type (even during feature foraging) for long runs, to prevent effortful switching. Our findings cannot be considered support for this, given the small costs involved with switching. Let us note that Beck, Hollingworth, and Luck (2012) reached similar conclusions using a different paradigm.

The Theory of Visual Attention (Bundesen, 1990; Bundesen and Habekost, 2008) may fare better than others in accounting for the observed data. Theory of Visual Attention differs from other theories in that two feature values can simultaneously be weighted proportionally. The theory can account for switching during foraging by relative weightings of two target types (e.g., green weight = .5; red weight = .5).

### Implications Regarding Priming of Attention Shifts

Research on priming of attention shifts (Jóhannesson & Kristjánsson, 2013; Kristjánsson & Campana, 2010; Lamy & Kristjánsson, 2013) shows how switching between different target types is effortful (Kristjánsson, Wang, & Nakayama, 2002; Maljkovic & Nakayama, 1994). A good strategy is therefore seemingly to stick to the same target type in a foraging task involving two or more targets. Clearly, observers should therefore repeatedly pick the same target type (Brascamp, Blake, & Kristjánsson, 2011; Chetverikov & Kristjánsson, 2015), but observers tend not to do this during feature foraging and a sizeable subset of observers do not do this even during conjunction foraging. This suggests that priming may only determine foraging when targets are cryptic (Dukas & Ellner, 1993; Nakayama, Maljkovic, & Kristjánsson, 2004) or perhaps when uncertainty regarding target identity is higher (Olivers & Meeter, 2006).

### Conclusions

Our results reveal both similarities and differences in foraging behavior across finger and gaze foraging. The results lend only partial support to proposals of overlap of eye movement control, motor control, and visual attention. All observers could switch easily between different target types during feature foraging, but many could also do this during conjunction foraging without notable costs, in particular during gaze foraging. Such rapid switching runs counter to key predictions of many theories of visual attention and WM.
